# α-Synuclein Genetic Variants Predict Faster Motor Symptom Progression in Idiopathic Parkinson Disease

**DOI:** 10.1371/journal.pone.0036199

**Published:** 2012-05-15

**Authors:** Beate Ritz, Shannon L. Rhodes, Yvette Bordelon, Jeff Bronstein

**Affiliations:** 1 Department of Epidemiology, University of California Los Angeles, Los Angeles, California, United States of America; 2 Department of Neurology, University of California Los Angeles, Los Angeles, California, United States of America; UCL Institute of Neurology, United Kingdom

## Abstract

Currently, there are no reported genetic predictors of motor symptom progression in Parkinson’s disease (PD). In familial PD, disease severity is associated with higher α-synuclein (*SNCA*) expression levels, and in postmortem studies expression varies with *SNCA* genetic variants. Furthermore, *SNCA* is a well-known risk factor for PD occurrence. We recruited Parkinson’s patients from the communities of three central California counties to investigate the influence of *SNCA* genetic variants on motor symptom progression in idiopathic PD. We repeatedly assessed this cohort of patients over an average of 5.1 years for motor symptom changes employing the Unified Parkinson’s Disease Rating Scale (UPDRS). Of 363 population-based incident PD cases diagnosed less than 3 years from baseline assessment, 242 cases were successfully re-contacted and 233 were re-examined at least once. Of subjects lost to follow-up, 69% were due to death. Adjusting for covariates, risk of faster decline of motor function as measured by annual increase in motor UPDRS exam score was increased 4-fold in carriers of the REP1 263bp promoter variant (OR 4.03, 95%CI:1.57–10.4). Our data also suggest a contribution to increased risk by the G-allele for rs356165 (OR 1.66; 95%CI:0.96–2.88), and we observed a strong trend across categories when both genetic variants were considered (p for trend  = 0.002). Our population-based study has demonstrated that *SNCA* variants are strong predictors of faster motor decline in idiopathic PD. *SNCA* may be a promising target for therapies and may help identify patients who will benefit most from early interventions. This is the first study to link *SNCA* to motor symptom decline in a longitudinal progression study.

## Introduction

Parkinson’s disease (PD) is a neurological condition characterized by progressive functional decline in motor function leading to disability and loss of quality of life [1]. Rate and severity of decline vary among patients but in all cases deterioration is inevitable as there are no current disease-modifying therapies that prevent or slow the neurodegenerative processes that cause PD [2]. More than a decade ago, clinical predictors of motor symptom progression were identified [3] including postural instability/gait disturbances, low Activities of Daily Living score, and dementia early in PD; since then only age at onset of symptoms has been added to this list [4]. One possible reason for the continuing paucity of information on risk factors for motor progression is that most PD patient cohorts with longitudinal data are comprised of non-representative groups of patients such as volunteers for clinical trials or referral patients at tertiary care centers. Such cohorts typically contain little information on non-clinical factors thereby presenting challenges for the identification of risk factors for progression beyond the most common clinical characteristics (such as age of symptom onset or motor subtype). Also, larger, mostly record-based studies are limited by the available outcomes (usually only comorbidities and mortality), typically lack of systematic motor symptom assessment, and generally have not collected lifestyle, environmental, or genetic risk factor data.

There are only a few population-based (community) studies publishing on motor progression using multiple motor symptom assessments over time, i.e., with longitudinally followed PD patients [3], [5], [6]. While one study [6] enrolled incident cases, two others [3], [5] enrolled prevalent cases with long (>5 years) mean duration of disease prior to baseline assessment thereby preventing observation of the early stages of disease progression and therefore perhaps overlooking critical factors involved in faster motor symptom decline [7]. Furthermore, these previous longitudinal studies started with fewer than 250 PD cases at baseline and all had only 50–60% of subjects remaining for follow-up assessment rendering them under-powered to examine all but the most common predictors of motor decline.

Recent publications have called for prospective cohorts of PD patients assembled early in disease [8] and clinico-genetic studies [9] to help unlock the heterogeneity of PD and its progression. Identifying genetic predictors may aid clinical decision-making, help target patients for treatment trials, and improve our understanding of disease processes. Thus far, genetic predictors of motor decline have not been reported in longitudinally followed incident PD cohorts, largely due to the limited number of such cohorts with biosamples.

Prior research has demonstrated a robust association between risk of PD occurrence and genetic variation in the *α-synuclein* (*SNCA*) gene [10]–[16]. *SNCA* has been a candidate gene for idiopathic PD ever since the localization of the PARK1 loci to the *SNCA* gene region in a large Italian kindred [17] and identification of SNCA protein in Lewy bodies [18]. By far the most frequently investigated genetic variant in *SNCA* is the microsatellite D4S3481 (*SNCA-*Rep1), located in the promoter, approximately 10 kb upstream of *SNCA*, and first reported in the Alzheimer literature [19]. While the first PD association study of *SNCA*-Rep1 [20] did not observed a difference in allele distribution between PD patients with a family history of PD and unaffected controls, it did find a significant difference in allele distribution between PD patients without a family history and unaffected controls. Two following studies [21], [22] observed a higher frequency of the 263 base pair (bp) repeat allele in PD cases compared with healthy controls. In 2006, a meta-analysis of 11 study populations including over 5000 subjects provided strong evidence that the 263bp allele was more frequent in cases increasing risk of PD (OR 1.43, 95% CI 1.22–1.69, dominant model) and that the 259bp allele was more frequent in controls decreasing risk of PD (OR = 0.85, 95% CI 0.76–0.96, dominant model); while the 261bp allele did not differ between PD cases and unaffected controls [10].

The linkage disequilibrium (LD) structure of *SNCA* suggests two distinct haplotype blocks, one covering an area from the 5′ end of the gene (the region of *SNCA*-Rep1) to exon 4 and the other covering from intron 4 to the 3′ region of the gene [23], [24]. While many studies have investigated haplotypes containing the *SNCA*-Rep1 promoter variant in conjunction with other single nucleotide polymorphisms (SNPs) in the 5′ region [10], [21], [24]–[32], use of promoter SNPs in place of or in addition to *SNCA*-Rep1 has not yet demonstrated improvement in risk estimation [10], [32]. In the 3′ region of *SNCA* most studies have investigated either rs356165 (first reported in Pals et al [26]; meta-analysis of 11 Caucasian studies, OR 1.32, 95%CI: 1.23–1.42 [33]) or rs356219 (first reported in Mueller et al [28]; meta-analysis of 18 Caucasian studies, OR = 1.28, 95%CI: 1.23–1.32 [33]). These two SNPs are in very high LD (D’ = 1, r2 = 0.962, LOD = 26.27 [34]) and either one uniquely identifies the two most common 3′ region haplotypes as reported in Pankratz et al [35]. In contrast, correlation between *SNCA*-Rep1 and rs356219 was found to be very low (r2 = 0.09) in a recent large study [36] supporting the hypothesis of two regions contributing to genetic risk of PD [37]. However, others hypothesized that the *SNCA*-Rep1 263bp allele arose on the haplotype background defined by the ‘G’ allele at rs356165 (or rs356219) thereby indicating a single genetic effect [23].

While findings from studies investigating *SNCA* 5′ and 3′ region genetic variants and age of onset of PD have been inconsistent [10], [16], [30], [31], [35], [38]–[43], there is data supporting biologic effects for genetic variants in *SNCA* possibly modifying the PD phenotype. Both *SNCA*-Rep1 [44], [45] and 3′ region alleles [46] have been associated with increased gene expression; and 3′ region risk alleles have been reported to correlate with higher plasma alpha-synuclein levels in PD patients [36]. Gene duplication [47], [48] and triplication [49], [50] in familial PD have been linked to increased mRNA expression levels [50]–[52] and to severity of disease [53], [54]. Furthermore, the α-synuclein protein product, a major component of Lewy bodies [18], likely contributes significantly to PD neurodegeneration and consequently to motor symptom decline. Therefore, in this large, longitudinally followed population-based incident PD cohort we investigate, for the first time, whether genetic variation in the *SNCA* gene, a known risk factor for PD onset, contributes to PD motor symptom decline.

## Methods

This study was approved by the human subjects committee of the University of Los Angeles (UCLA). Subjects participated in the study after written informed consent was obtained; prospective participants and their families/guardians were asked to discuss the consent documents; consent on behalf of participants deemed incapable of consenting was obtained from those determined to be legal proxies for the patient.

### Study Population

This longitudinal patient cohort is derived from a case-control study that enrolled PD patients and population-based controls between 2001 and 2007 in Central California. Recruitment methods [55], [56] and case definition criteria [57] have been described in detail elsewhere. Briefly, of 1,167 PD patients initially invited, 604 did not meet eligibility criteria primarily due to having a first physician diagnosis of PD more than 3 years prior to baseline. Of the 563 eligible patients, 473(84%) were examined by our movement disorder specialists (JB and YB) at least once; 104 did not meet published criteria for idiopathic PD [58] and 6 had incomplete data. The remaining 363 idiopathic PD patients compose the PD cohort that is the basis of this longitudinal follow-up investigation.

At first re-contact 83(22.9%) patients were deceased, 25(6.9%) withdrew, 9(2.5%) could not be found, and 4(1.1%) were too ill to participate. For follow-up, 242(66.6%) cases were successfully re-contacted with 6 patients participating by mail/phone only (no in-person follow-up exam and therefore not contributing to this analysis) and 3 patients re-classified by us during follow-up as not having idiopathic PD. Of 233 patients re-examined and confirmed to have idiopathic PD, 178(76.4%) were seen twice and 55 were seen only once during follow-up (21 were deceased prior to the second exam and 14 are still pending second exams, 8 were too ill to participate, 4 could not be not located, 3 withdrew, and 5 participated by mail/phone only). For these analyses, 232 of the 233 patients with at least one follow-up had *SNCA* genotype data available. All patients completed an interview to collect demographic and risk factor data; and provided blood samples for DNA.

### Assessment of PD Motor Symptom Progression

Baseline and follow-up Unified Parkinson’s Disease Rating Scale (UPDRS) [59] exams were performed at a clinic near the patient’s residence or at that residence if the patient was unable to travel. Patients were examined off PD medications (i.e. overnight medication withdrawal prior to exam) whenever possible (82% of patients were off medication for baseline exam, 80% for follow-up exams). For patients and time points without an off exam, we estimated the off exam score by adding to the patient’s on exam score the difference of the study population’s mean off- and mean on-scores. At each exam 6% of PD patients were unable to perform one or more motor UPDRS exam items (e.g. patients unable to walk due to a missing lower limb); we replaced missing exam items either assuming no change from baseline to follow-up for patients with available baseline data or assuming the population mean for patients with neither baseline nor follow-up values available, an approach likely to bias our estimates towards the null and therefore a conservative approach.

Annual rate of change in UPDRS motor score was calculated as the difference of the last follow-up and the baseline motor scores divided by the interval of time (in years) between exams. We did not assume a linear association between the *SNCA* genetic variants and rates of motor progression since our analyses would be too crude to predict actual levels of synuclein. Therefore, given that a 5 points/year change in motor UPDRS has been reported for early untreated PD patients in placebo arms of clinical trials [2] and is considered a clinically relevant change when assessing improvement due to treatment [60], we defined “fast” motor symptom decline as a 5 or more point increase in the annual rate of change.

### Determination of SNCA Variants

*SNCA* genetic variants were genotyped according to previously published methods [30], [61]. The *SNCA* microsatellite Rep1 (D4S3481) was genotyped using fluorescent-labeled forward and reverse primers designed to the α-synuclein promoter locus (accession no. U46895), Fam5′-CCT GGC ATA TTT GAT TGC AA-3′ and 5′-GAC TGG CCC AAG ATT AAC CA-3′. Detection and analysis was performed on an ABI 3730 DNA Analyzer (Applied Biosystems, Inc.) with Genemapper software. *SNCA* rs356165 in the 3′ untranslated region (3′UTR) was genotyped on an ABI 7900 Genome Analyzer using ABI Taqman® chemistry.

### Statistical Analysis

Hardy-Weinberg equilibrium was assessed for each variant using Mendel 10.0 [62]. Logistic regression analyses (SAS 9.1.3, SAS Institute, Cary, NC) were employed to estimate odds ratios (OR) and 95% confidence intervals (CI) adjusting for age at PD diagnosis (continuous), gender (male/female), smoking status (never/ever), duration of time between diagnosis and baseline assessment (in years), and baseline UPDRS motor score. Subgroup analyses were performed limited to (1) subjects with off-medication exams only to investigate the influence of estimating missing off exam scores from on-medications motor exam scores (excludes 83 subjects who were unable to be assessed off PD medications at either baseline or follow-up) and limited to (2) subjects with complete UPDRS data at both baseline and follow-up exams to investigate the influence of replacing missing UPDRS motor exam items as described above (excludes 26 subjects who were unable to perform one or more motor UPDRS exam items at either baseline or follow-up, e.g. patients unable to walk due to a missing lower limb).

The repeat lengths of *SNCA*-Rep1 were analyzed in the manner originally presented by Maraganore et al [10]: a dominant genetic model comparing 263/263 and 263/X vs. X/X, where X is either the 259 or the 261 allele; and a dominant genetic model comparing 259/259 and 259/X vs. X/X, where X is either the 261 or the 263 allele. The *SNCA* SNP rs356165 was analyzed under an additive (or allele dosage) genetic model similarly to prior studies [28], [30], [31]. Assuming that the 5′ *SNCA*-Rep1 and the 3′ rs356165 regions represent independent contributors to genetic risk of PD [31], [36], [37], and possibly also to progression of motor symptoms, we investigated potential statistical interaction between *SNCA*-Rep1 263bp allele and the rs356165 ‘G’ allele. P-values presented are uncorrected; for multiple comparisons considerations, four tests were performed and a p-value of 0.0125 was considered the experiment-wide significance level. Sensitivity analyses were performed to assess model robustness to additional possible covariates and the outcome cut-point.

## Results

The characteristics of PD patients in this report are summarized in tables 1 and 2. We followed patients on average for 5.1 (std dev 2.2) years and categorized 35 (15.1%) patients as “fast progressors” for motor symptom decline as measured by the motor UPDRS. Fast progressors were older at time of PD diagnosis, had a lower UPDRS motor exam score at baseline, a shorter follow-up, and had lower baseline bradykinesia, rigidity, and tremor subscores (table 2), but did not differ from slow progressors in other risk factors including predominant motor type (akinetic [63]: 74% and 81%, respectively; p-value = 0.24). Deceased patients were older, had a higher baseline UPDRS motor score, higher bradykinesia and gait/balance subscores, a lower Mini Mental State Exam (MMSE) [64], and a higher Geriatric Depression Score (GDS) [65] than both slow and fast progressors. Participants lost to follow-up but not deceased were not different from slow progressors on demographics or risk factors. Neither deceased patients nor those lost to follow-up differed from slow progressors on *SNCA*-Rep1 or rs356165 genetic variants (table 3). Finally, although carriers of the 263bp *SNCA*-Rep1 risk allele were on average younger (64 years) than non-carriers (67 years) at time of PD diagnosis, this difference was not formally statistically significant (p>0.10).

**Table 1:** Demographic characteristics of Parkinson’s disease cohort members at baseline exam (2001–2007).

	Slow Progressor	Fast Progressor	p*	Deceased	p§	Lost to Follow-up	p†
**All (n)**	197	35		83		44	
**Age at PD Diagnosis (mean±SD)**	66.4±9.9	69.6±8.6	0.07	73.9±6.8	<0.01	64.9±13.4	0.46
**Age Range of Subjects**	35–88	50–83		47–88		34–84	
**Female (%)**	40.6	48.6	0.38	47.0	0.32	40.9	0.97
**Never Smokers (%)**	55.8	40.0	0.08	51.8	0.54	47.7	0.33
**Positive Family History of PD (%)**	14.2	14.3	0.99	13.2	0.83	20.5	0.30
**Non-Caucasian Race/Ethnicity (%)**	18.8	22.9	0.57	18.1	0.89	20.4	0.80
**< = 12 year of school (%)**	39.2	33.3	0.62	42.2	0.74	55.6	0.21

Abbreviations: PD  =  Parkinson’s disease; SD  =  standard deviation.

* P-value for chi-square test or t-test of fast vs. slow progressors.

§ P-value for chi-square test or t-test of deceased participants vs. slow progressors.

† P-value for chi-square test or t-test of lost participants vs. slow progressors.

**Table 2:** Phenotypic characteristics of Parkinson’s disease cohort members at baseline exam (2001–2007) and follow-up (2008–2010).

	Slow Progressor	Fast Progressor	p*	Deceased	p§	Lost to Follow-up	p†
**All (n)**	197	35		83		44	
**Duration of PD prior to baseline**							
** mean±SD (years)**	1.84±1.3	2.01±1.6	0.50	2.08±1.35	0.16	1.88±1.16	0.86
** range**	0–7.1	0–6.7		0–4.9		0–4.5	
**Interval between exams**							
** mean±SD (years)**	5.20±2.2	4.26±2.1	0.02	n.a.	n.c.	n.a.	n.c.
** range**	0.85–9.1	1.14–8.7		n.a.		n.a.	
**Baseline UPDRS motor score**							
** mean±SD (points)**	20.5±9.7	15.8±7.5	0.01	27.0±13.6	<0.01	20.3±10.5	0.89
** range**	4–49	3–31		5–68.6		3–51	
**Mean annual change in UPDRS motor score**							
** mean±SD (points per year)**	1.73±1.5	6.95±3.4	<0.01	n.a.	n.c.	n.a.	n.c.
** range**	0–4.94	5–24.6		n.a.		n.a.	
**Baseline PD Subscores** **							
** Bradykinesia (mean±SD)**	6.89±4.0	4.96±3.4	0.01	9.31±5.3	<0.01	6.80±4.3	0.89
** Rigidity (mean±SD)**	3.36±2.4	2.40±1.5	<0.01	3.86±3.1	0.19	3.48±2.3	0.77
** Tremor (mean±SD)**	1.80±1.9	1.20±1.1	0.01	2.02±2.3	0.45	1.39±1.6	0.17
** Gait/Balance (mean±SD)**	2.92±2.0	2.81±2.1	0.78	5.18±3.3	<0.01	3.43±2.6	0.23
**Baseline MMSE** §§	28.2±2.4	27.8±1.9	0.44	26.5±3.0	<0.01	27.9±2.5	0.61
**Baseline GDS^††^**	3.26±3.4	3.31±3.0	0.94	4.25±2.8	0.01	3.44±3.1	0.75

Abbreviations: SD  =  standard deviation; PD  =  Parkinson’s disease; yrs  =  years; n.a.  =  not applicable; n.c.  =  not calculated; UPDRS  =  Unified Parkinson’s Disease Rating Scale; MMSE  =  Mini Mental State Exam; GDS  =  Geriatric Depression Score.

* P-value for t-test of fast vs. slow progressors.

§ P-value for t-test of deceased participants vs. slow progressors.

† P-value for t-test of lost participants vs. slow progressors.

** PD subscores (bradykinesia, rigidity, tremor, gait/balance) calculated according to Louis (ref 3).

§§ Mini Mental State Exam score according to Folstein (ref 64).

† ^†^Geriatric Depression Score according to Yesavage (ref 65).

**Table 3:** SNCA Rep1 (D4S3481) and rs356165 genotypes of Parkinson’s disease cohort members.

	Slow Progressor	Fast Progressor	p*	Deceased	p§	Lost to Follow-up	p†
**All (n)**	197	35		83**		44	
**SNCA Rep1 genotypes, n(%)**							
** 259/259**	20 (10.2)	1 (2.9)		11 (13.6)		1 (2.3)	
** 259/261**	75 (38.1)	9 (25.7)		30 (37.0)		23 (52.3)	
** 259/263**	5 (2.5)	4 (11.4)		6 (7.4)		3 (6.8)	
** 261/261**	82 (41.6)	15 (42.9)		30 (37.0)		13 (29.6)	
** 261/263**	12 (6.1)	6 (17.1)		4 (5.0)		4 (9.1)	
** 263/263**	3 (1.5)	0 (0.0)	0.01	0 (0.0)	0.33	0 (0.0)	0.12
**SNCA rs356165**							
** AA**	63 (32.0)	8 (22.9)		29 (35.4)		14 (31.8)	
** AG**	101 (51.3)	16 (45.7)		40 (48.8)		22 (50.0)	
** GG**	33 (16.7)	11 (31.4)	0.11	13 (15.8)	0.86	8 (18.2)	0.97

Abbreviations: SNCA  =  alpha-synuclein.

* P-value for chi-square test of fast vs. slow progressors.

§ P-value for chi-square test of deceased participants vs. slow progressors.

† P-value for chi-square test of lost participants vs. slow progressors.

** One deceased subject had a genotype of 257/259 and one subject has not been genotyped for SNCA Rep1 and rs356165.

In logistic regression analyses adjusting for age, gender, smoking, duration of time between diagnosis and baseline assessment, and baseline UPDRS motor score, we observed no association between the *SNCA-*Rep1 259 allele and progression under a dominant genetic model although the odds ratio is in the expected (“protective”) direction (table 4). In contrast, PD patients carrying at least one 263bp allele in *SNCA*-Rep1 exhibited 4-fold higher odds of fast progression (OR = 4.03; 95% CI: 1.57–10.4; table 5). Separately, the G allele of *SNCA* rs356165 increased risk of faster progression 60% (one copy) or 270% (two copies) under an additive genetic model (table 6), albeit this association did not reach statistical significance. Effect estimates were robust to adjustment for education, minority status, PD family history, and bradykinesia, rigidity, tremor, and gait/balance subscores.

**Table 4:** Logistic regression odds ratio (and 95%CI) for faster progression* by *SNCA* Rep1 genotypes defined by the 259 base pair allele vs others (dominant model).

	Primary Analysis				Subgroup #1				Subgroup #2			
	Slow	Fast	OR(95%CI)	p†	Slow	Fast	OR(95%CI)	p†	Slow	Fast	OR(95%CI)	p†
**Subjects (n)**	197	35			125	24			174	32		
**259 non-carriers (%)** §	49.2	60.0	1.00(reference)		47.2	62.5	1.00(reference)		50.6	59.4	1.00(reference)	
**259 carriers (%)** §	50.8	40.0	0.76(0.35−1.65)		52.8	37.5	0.60(0.23–1.54)		49.4	40.6	0.88(0.39–1.98)	
				0.486				0.286				0.753

All analyses are adjusted for age at diagnosis (continuous), gender (male/female), smoking status (never/ever), duration of time between PD diagnosis and baseline assessment (in years), and baseline UPDRS motor score.

Abbreviations: SNCA  =  alpha-synuclein; OR  =  odds ratio; CI  =  confidence interval; p  =  p-value; n  =  number.

Subgroup #1, subjects with off-medication exams only.

Subgroup #2, subject with complete UPDRS data at both baseline and follow-up exams.

* Faster progression defined as ≥5 point change in motor UPDRS annually.

§ 259 non-carriers include genotypes 261/261, 261/263, or 263/263; 259 carriers include genotypes 259/259, 259/261 or 259/263.

† P-value the additive (or allele dosage) genetic model.

**Table 5:** Logistic regression odds ratio (and 95%CI) for faster progression* by *SNCA* Rep1 genotypes defined by the 263 base pair allele vs others (dominant model).

	Primary Analysis				Subgroup #1				Subgroup #2			
	Slow	Fast	OR(95%CI)	p†	Slow	Fast	OR(95%CI)	p†	Slow	Fast	OR(95%CI)	p†
**Subjects (n)**	197	35			125	24			174	32		
**263 non-carriers (%)** §	89.9	71.4	1.00(reference)		92.8	66.7	1.00(reference)		89.7	68.8	1.00(reference)	
**263 carriers (%)** §	10.2	28.6	4.03(1.57–10.4)		7.2	33.3	9.00(2.63–30.8)		10.3	31.2	4.53(1.69–12.1)	
				0.004				<0.001				0.003

All analyses are adjusted for age at diagnosis (continuous), gender (male/female), smoking status (never/ever), duration of time between PD diagnosis and baseline assessment (in years), and baseline UPDRS motor score.

Abbreviations: SNCA  =  alpha-synuclein; OR  =  odds ratio; CI  =  confidence interval; p  =  p-value; n  =  number.

Subgroup #1, subjects with off-medication exams only.

Subgroup #2, subject with complete UPDRS data at both baseline and follow-up exams.

* Faster progression defined as ≥5 point change in motor UPDRS annually.

§ 263 non-carriers include genotypes 261/261, 261/259, or 259/259; 263 carriers include genotypes 263/263, 261/263 or 259/263.

† P-value the additive (or allele dosage) genetic model.

**Table 6:** Logistic regression odds ratio (and 95%CI) for faster progression* by *SNCA* rs356165 genotypes (additive model).

	Primary Analysis				Subgroup #1				Subgroup #2			
	Slow	Fast	OR(95%CI)	p§	Slow	Fast	OR(95%CI)	p§	Slow	Fast	OR(95%CI)	p§
**Subjects (n)**	197	35			125	24			174	32		
**AA (%)**	32.0	22.9	1.00(reference)		27.2	25.0	1.00(reference)		31.0	18.7	1.00(reference)	
**AG (%)**	51.3	45.7	1.66(0.96–2.88)		56.0	41.7	1.57(0.77–3.19)		52.3	46.9	1.96(1.07–3.62)	
**GG (%)**	16.7	31.4	2.76(1.57–4.84)		16.8	33.3	2.45(1.20–4.99)		16.7	34.4	3.85(2.09–7.10)	
				0.071				0.201				0.027

All analyses are adjusted for age at diagnosis (continuous), gender (male/female), smoking status (never/ever), duration of time between PD diagnosis and baseline assessment (in years), and baseline UPDRS motor score.

Abbreviations: SNCA  =  alpha-synuclein; OR  =  odds ratio; CI  =  confidence interval; p  =  p-value; n  =  number.

Subgroup #1, subjects with off-medication exams only.

Subgroup #2, subject with complete UPDRS data at both baseline and follow-up exams.

* Faster progression defined as ≥5 point change in motor UPDRS annually.

§ p-value for the additive (or allele dosage) genetic model.

In subgroup analyses restricted to patients with only off medication UPDRS exams or subjects with complete UPDRS data, the estimated effects were similar to or stronger than those for the full study sample (tables 4, 5, 6) although the confidence intervals were wider due to smaller sample sizes. Our results were relatively insensitive to lowering the cut-point for fast progression (e.g. top quartile rate of change *vs*. others, *SNCA-*Rep1 OR = 2.66; 95% CI: 1.14–6.19). Finally, although we did not observe statistical interaction, patients with the ‘GG’ risk genotype at rs356165 and without the *SNCA*-Rep1 263bp risk allele exhibited a smaller increase in risk of faster progression than patients with the ‘GG’ risk genotype and with at least one copy of the *SNCA*-Rep1 263bp risk allele; additionally, we observed a strong trend across categories of the combined genetic variants (table 7; p-value for trend 0.002).

**Table 7:** Logistic regression odds ratio (and 95%CI) for faster progression* by *SNCA* Rep1 263bp allele and rs356165 genotypes.

	Rep1 genotypes 259/259, 259/261, 261/261			Rep1 genotypes 259/263, 261/263, 263/263			
rs356165	Slow n (%)	Fast n (%)	OR (95%CI)	Slow n (%)	Fast n (%)	OR (95%CI)	P- trend§
**AA**	63 (32.0)	7 (20.0)	1.00 (reference)	0 (0.0)	1 (2.9)	n.c.	
**AG**	88 (44.7)	11 (31.4)	1.14 (0.40–3.22)	13 (6.6)	5 (14.3)	4.01 (1.02–15.8)	
**GG**	26 (13.2)	7 (20.0)	2.40 (0.72–8.06)	7 (3.5)	4 (11.4)	6.29 (1.26–31.5)	
							0.002

All analyses are adjusted for age at diagnosis (continuous), gender (male/female), smoking status (never/ever), duration of time between PD diagnosis and baseline assessment (in years), and baseline UPDRS motor score.

Abbreviations: SNCA  =  alpha-synuclein; OR  =  odds ratio; CI  =  confidence interval; p  =  p-value; n  =  number; n.c.  =  not calculated.

Subgroup #1, subjects with off-medication exams only.

Subgroup #2, subject with complete UPDRS data at both baseline and follow-up exams.

* Faster progression defined as ≥5 point change in motor UPDRS annually.

§ a P-value for trend was generated using an ordered categorical variable combining the two genetic variants as indicated by the table.

## Discussion

Heterogeneity in clinical presentation is widely acknowledged in PD [8] and the rate of disease progression and motor symptom decline is known to vary strongly [66]. The α-synuclein protein is a major component of Lewy bodies and participates in the molecular pathogenesis of PD. Mutations and multiplications of the *SNCA* gene cause familial parkinsonism and genetic variations are recognized as risk factors for idiopathic PD [50]. Here we present the first evidence that *SNCA* is an important and strong predictor of faster motor symptom decline in idiopathic PD: we observed a 4-fold higher risk of PD for carriers of the *SNCA*-Rep1 263bp allele and, when considering both Rep1 263bp and rs356165 ‘G’ allele, we observed a strong trend for faster motor decline across categories of the combined genetic variants.

*SNCA*-Rep1 allelic variants and 3′UTR variants (including rs356165) are correlated with *SNCA* mRNA expression in human postmortem brain tissues [46], [67]. Furthermore, in familial parkinsonism with gene duplication or triplication increased mRNA expression has been linked to severity of disease (e.g. younger onset, faster decline, cognitive impairment) [50]. Thus, evidence that these PD-associated *SNCA* variants have functional consequences is accumulating rapidly. In our population-based idiopathic PD cohort, both the *SNCA-*Rep1 promoter and 3′UTR risk variants in combination with the promoter predicted faster motor symptom decline and these findings suggest that α-synuclein levels in idiopathic PD are strongly associated with progression as well as with disease risk.

Apart from clinical trials of mostly younger onset patients [68]–[73], and hospital-based studies of prevalent cases [4], [66], [74], only three prior population-based studies conducted in New York [3], Norway [5], and the UK [6] collected longitudinal UPDRS data for evaluating PD motor progression. All three population-based studies were limited by follow-up sample size (<150 subjects remaining after 4 years or more); whereas our study has 232 subjects at on average 5.1 years of follow-up, and thus is the largest population-based longitudinal study of PD motor symptoms in the literature to date. Additionally, two studies [3], [5] assessed only prevalent cases with long mean duration of disease prior to baseline (6.8–9.1 years), thus missing early stages of disease progression, a potentially critical time for neurodegeneration of the dopamine system [66]. Faster motor decline in early disease is consistent with an early accelerated dopaminergic neurodegeneration and an exponential demise of neurons shortly after PD onset that levels off over time [75]. This early stage of PD might be the most sensitive and effective period for interventions aimed at slowing disease progression.

Our PD cohort is population-based and, thus, we expect it to adequately represent the range of disease phenotypes and treatment protocols found in the general U.S. PD population. Our study design also ensured representativeness of our patients to idiopathic PD patients seen in the health care system in general, different from more common PD progression studies that assembled homogeneous groups of very select patients from clinical trials or tertiary care facilities. The average age and age range of our PD patient cohort at diagnosis (68.3 years; range 34–88) is as expected in population based studies of PD; and similar to what was reported in the longitudinal study of incident PD in the UK (70.1 years; range 37–94) [6], [76], and to the age range in both prevalence progression studies in NY (range 25–93) [3] and Norway (range 27–85) [77]. Moreover, the distributions of *SNCA* Rep1 and rs356165 variants observed in our cohort of PD cases are very similar to those observed for PD cases in studies indexed by PDGene (www.pdgene.org [33]). Thus, the results observed in our population-based cohort are more likely to be generalizable to the U.S. PD population than results of placebo arms of clinical trials that limit enrollment to a select age range or stage of disease.

Consistent with observations from a recent longitudinal imaging study [78] the motor symptoms of our PD patients age 66 and younger declined at a slower rate than those of our patients age 67 and older (2.13 points/year vs. 2.80 points/year, p = 0.043). Adjusting for age demonstrated that our observed genetic associations are independent of age. In fact, age-stratified analyses (< = 66 and >66), while underpowered, produced similar odds ratio estimates as the combined sample.

Due to the logistics of a population based study, we were unable to follow our patients on an annual or more regular basis, but our average annual rate of change measure accounts for differences in follow-up intervals. Moreover, our slow progressors were followed for a slightly longer interval thereby somewhat minimizing the extent to which slow progressing subjects were misclassified due to follow-up duration, since patients are less likely to improve once reaching a more advanced state of motor symptom decline. Finally, analysis limited to subjects with an interval from baseline to follow-up of 3 years or more (mean 5.82 years follow-up) produced odds ratios similar to analyses of the full sample (e.g. OR_Rep1 263_ = 4.05, 95%CI: 1.42–11.6).

We lost a third of all patients between baseline and follow-up, mostly because participants had died. Other population-based studies of PD have similar or greater losses at follow-up: NY [3] lost 45% at 4 years, Norway [5] lost 38% at 4 years, and UK [6] 48% at 6.6 years of follow-up. Of our subjects lost to follow-up, 69% had died during follow-up; this is similar to the only other study of incident PD cases [6] where 64% of lost subjects had died during follow-up. Relevant to our findings, when we examined the genetic variants of *SNCA* in those lost due to death and lost due to other reasons, we observed no differences from the comparison group of slow progressors (p>0.10).

Unlike many longitudinal PD studies, we attempted to examine all patients in a practically defined off PD medication state, but this was not achieved in about 20% of exams. While this could have caused non-differential outcome misclassification with regard to genetic factors and an underestimation of the rate of progression and the effect size, we observed an overall mean change in UPDRS motor score of 2.52 points/year (average follow-up 5.1 years) which is very similar to the rate observed by the only other population-based incident study that estimated a mean change of 2.24 points/year (average follow-up 6.6 years) albeit this rate represents results from “on-on” exams [6].

Since motor symptom severity predicts increased mortality in PD independent of age and disease duration [79], identifying genetic predictors of faster motor decline is critical to pinpointing biological mechanisms as targets for therapies and identifying patients who will most benefit from early interventions. While replication of our results in similarly well-characterized population-based incidence PD cohorts that have been longitudinally followed is still needed, our findings strongly suggest that α-synuclein and related pathogenic pathways have great promise as potential disease modifying and therapeutic targets.
